# Categorizing Patients in a Forced-Choice Triad Task: The Integration of Context in Patient Management

**DOI:** 10.1371/journal.pone.0005881

**Published:** 2009-06-11

**Authors:** Sarah L. Devantier, John Paul Minda, Mark Goldszmidt, Wael Haddara

**Affiliations:** 1 Department of Psychology, The University of Western Ontario, London, Ontario, Canada; 2 The Schulich School of Medicine & Dentistry, The University of Western Ontario London, Ontario, Canada; University Paris 7, France

## Abstract

**Background:**

Studies of experts' problem-solving abilities have shown that experts can attend to the deep structure of a problem whereas novices attend to the surface structure. Although this effect has been replicated in many domains, there has been little investigation into such effects in medicine in general or patient management in particular.

**Methodology/Principal Findings:**

We designed a 10-item forced-choice triad task in which subjects chose which one of two hypothetical patients best matched a target patient. The target and its potential matches were related in terms of surface features (e.g., two patients of a similar age and gender) and deep features (e.g., two diabetic patients with similar management strategies: a patient with arthritis and a blind patient would both have difficulty with self-injected insulin). We hypothesized that experts would have greater knowledge of management categories and would be more likely to choose deep matches. We contacted 130 novices (medical students), 11 intermediates (medical residents), and 159 experts (practicing endocrinologists) and 15, 11, and 8 subjects (respectively) completed the task. A linear mixed effects model indicated that novices were less likely to make deep matches than experts (*t*(68) = −3.63, *p* = .0006), while intermediates did not differ from experts (*t*(68) = −0.24, *p* = .81). We also found that the number of years in practice correlated with performance on diagnostic (*r* = .39, *p* = .02), but not management triads (*r* = .17, *p* = .34).

**Conclusions:**

We found that experts were more likely than novices to match patients based on deep features, and that this pattern held for both diagnostic and management triads. Further, management and diagnostic triads were equally salient for expert physicians suggesting that physicians recognize and may create management-oriented categories of patients.

## Introduction

The topic of clinical reasoning in medicine has been studied extensively [1]–[4]. The predominant focus of this literature has related to the process of diagnosis, but it is well recognized by practitioners in the field that clinical reasoning encompasses a far wider set of tasks than simply arriving at the correct diagnosis. Physicians also use contextual cues, patient preferences, and the best available evidence to make decisions about how to manage patients and how to help patients manage their disease or diseases [5]. For example, consider a patient with poorly controlled diabetes where all of the biomedical evidence suggests that the appropriate therapy is multiple daily injections of insulin. Now imagine that this same patient lives alone, has minimal family support, and has severe crippling arthritis such that she could not inject insulin even if she were motivated to do so. Recognizing this conflict between the best evidence and the context in which the patient finds him or herself and working with the patient to identify a safe and feasible management plan is a crucial component of the reasoning activity involved in caring for this patient. In fact, the diagnosis was never at issue in this patient encounter.

The extent to which physicians use information such as in the above example when reasoning and thinking about patients has received little study. We suggest that physicians recognize specific diagnostic and management categories of patients, and may classify a patient as belonging to one or more categories (diagnostic, treatment, or management) and may interact with the patient accordingly. With regard to the management categories, we further argue that, unlike diagnostic categories, which are largely taxonomic, management categories are examples of “goal-derived” or “ad-hoc” categories [6], [7] that are acquired through clinical experience. As physicians engage in the practice of medicine, they need to satisfy a variety of goals and a physician may group patients together according to how s/he might go about accomplishing those goals and managing those patients. We also argue that expertise in management does not necessitate expertise in diagnosis and that expertise in these two domains may advance along different trajectories as physicians progress through medical school, residency, and practice. Finally, we predict that if expertise in management comes from clinical experience, then expertise in management should correlate with clinical experience.

### Medical Reasoning

Though generally focused on the process of arriving at a diagnosis, there has been a large amount of research on medical reasoning. There are two main streams of research that we will address here: (1) the effects of experience, and (2) the effects of context, on medical reasoning. There is currently some debate over the organization of expert physicians' knowledge: through specific exemplars of patients, or around more generic illness scripts. Norman, Brooks, and colleagues [3], [4], [8] suggest that expert physicians are more successful at diagnosis than novices and intermediates because they recall relevant and specific examples of patients from their own experience, while novices and intermediates use more general illness scripts to recall the symptoms and course of an illness. They suggest novices and intermediates rely on these scripts because they do not have enough personal experience with a sufficient number of patients to create exemplar memories different illnesses. As medical students gain experience with different types of patients, they will begin moving away from illness scripts towards recall of particular instantiations of a disease, particularly for those illnesses with which they have the most experience. This hypothesized trajectory of expertise, from the formulaic performance of novices to the instance-based performance of experts, parallels the general treatment of expertise in the cognitive literature (e.g., [9]).

Conversely, Boshuizen, Schmidt, and colleagues (e.g., [10], [11]) suggest that novices and intermediates tend to recall a specific, recently seen patient, leaving them susceptible to exceptional circumstances. This suggests that novices may rely on an availability heuristic, and that the memory is based on recency or exceptionality, rather than clinical relevance. Experts, on the other hand, seem to rely on general illness scripts (e.g., the representativeness heuristic) for particular diseases in which irrelevant or exceptional information is filtered out. Researchers agree, however, that novices begin by learning about basic biomedical information and causal networks that explain the cause and consequences of diseases. As physicians gain more experience with patients, they gradually move away from using only basic science information into more integrated disease representations, combining basic science information with contextual information, allowing them to effectively manage patients [3], [4], [8], [10], [11].

Contextual information–information that describes the patient's living conditions and can include things such as age, sex, medical history, living environment, occupation, marital status, risk behaviors, etc. – can provide physicians with information that is important in determining the most appropriate treatment for their patients. In the example at the beginning of this paper, a patient has no home support, and is unable to check her own blood sugar levels or to inject insulin, both of which are necessary if a patient is to be placed on injectable insulin. This contextual information is essential for creating a successful management plan, since medication a patient cannot take (or takes incorrectly) will not help her disease.

Contextual information can allow physicians to reach a diagnosis more quickly and accurately. For example, Custers et al. [10], found that expert doctors were better at diagnosing disorders when given contextual information such as age, gender, medical history, occupation, etc. Novice and intermediate subjects tended to think about patients the same way with or without contextual cues. Verkoeijen, Rikers, Schmidt, Van De Wiel and Kooman [12], also found that context allowed physicians to more quickly and accurately diagnose patients, but that experts benefitted most from the addition of contextual information, suggesting that experts were most able to recognize the importance of context, or were most able to integrate context into their diagnoses. Looking at the illness scripts described by physicians and students for twenty common illnesses, novices were more likely to mention biomedical and basic science information, while experts reported little biomedical information and more contextual information [10]. Even for intermediate subjects (6^th^ year medical students), there was little integration of contextual information into their illness scripts, suggesting that context integration may take many years.

The progression in physicians' ability to utilize contextual information has been shown in other studies as well. In a study of the differences in how physicians go about prescribing mediations to patients, Higgins and Tully [13] found that novice prescribers tended not to consider context, and simply focused on the disease itself when prescribing medications. Intermediates were more patient focused than novices and involved the patient through discussion when considering prescription alternatives; however, there was still little use of context in their decisions. Finally, experts tended to focus on context when prescribing medications, considering the patient's life outside of the hospital as well as the disease characteristics of the patient. This study reveals a shift in how doctors think about patient management from objective, logical and distant, considering only the features of the illness as novices, to a patient-focused consideration of the context of the illness, as well as the illness itself, as experts.

Context is an important part of patient management, and based on the above findings, we expect that physicians' ability to integrate contextual information into their diagnoses and management plans should increase with experience. Further, the presence of contextual information should aid expert physicians more than it does less experienced subjects. Because experts are more likely to utilize contextual information, we believe experts should be more likely than novices or intermediates to group patients together whose contextual information has a similar impact on how they should be managed, even if that contextual information does not share surface similarities.

### Expert Thinking

Given that research on thinking and reasoning in medicine has focused nearly exclusively on the diagnostic process, we turned to research on thinking and expertise in general in order to develop an appropriate means of verifying and investigating medical expertise in patient management. The study of expert thinking is often carried out by investigating the problem-solving abilities of a specific kind of expert, such as experts in chess [14]–[16] or physics [17]–[19]. Of particular interest to our investigation is the finding that experts can often ignore or suppress attention to the surface features of a problem and attend instead to deep, solution-relevant features of a problem.

In an influential paper, Chi et al. [17] asked physics Ph.D. students (experts) and undergraduate students (novices) to sort 24 physics problems into groups and to explain the reasons for their groupings. Novices generally sorted the problems on the basis of surface features. That is, they grouped problems according to the literal physics terms mentioned in the problem and the physical configuration described in the problem. Experts, on the other hand, sorted their problems on the basis of deep features that were related to the major physics principles governing the solution of each problem. This suggests that experts accessed existing schemata and they used their knowledge of physics to create a solution-oriented sorting. Since the problems were sorted according to these categories, it also suggests that these categories would likely be accessed when deciding how to solve a problem.

Related research has investigated expert classification in naturalistic domains, such as tree classification and fish classification [20], [21]. For example, Medin et al. [20] asked a variety of experts to sort trees into different groups and found that taxonomists tended to group trees by phylogenetic factors, whereas landscape architects and maintenance workers grouped trees in terms of specific, shared goals (e.g., weed trees, ornamental flowering trees, etc.). Medin et al. also found that these classifications were dynamic and depended on specific task demands. When making inferences and inductions, the landscape architects also demonstrated sophisticated knowledge of phylogenetic factors. In related research, Shafto and Coley [21] found that undergraduates (novices) and commercial fisherman (experts) differed in how they classified fish. Generally, novices sorted on the basis of appearance and surface features whereas experts often sorted on the basis of commercial and behavioral factors. Experts also subdivided the fish on the basis of ecological niche. These studies suggest a general effect for expert-level classification: Experts can appreciate the deep features and contextual aspects of an object/entity and can base classification on those deep features. Furthermore, this research suggests that many of these deep features are related to specific goals of the expert at the time of sorting.

These results have implications for the study of how physicians think about patient management. For example, in the same way that a landscape architect or a physics Ph.D. student can perceive the deep structure related to the solution or use of a given entity/object, so too might an expert physician be able to perceive deep structures related not only to diagnosis but also to the management of their patients. Perceiving this deep structure could assist the expert physician in making decisions about how to treat a patient, how to interact with a patient, whether or not to follow up with a patient for compliance, etc. We argue that all of these things are central to being a good physician, but this kind of decision-making is not typically the focus of investigation and research in medicine.

### Forced-Choice Triad Task

An initial goal for our research was to develop a task that was sensitive to expertise differences in diagnostic reasoning as well as reasoning about patient management. To do this, we turned to a forced-choice triad task that is commonly used in cognitive psychology [22]–[26]. Forced-choice triad tasks are popular because they allow subjects to focus on fewer problems at a time than sorting tasks discussed above, and can be completed quickly [27].

As a way to illustrate the forced-choice triad task, consider the following trivial example. Suppose one is shown a RED APPLE as a target and then shown a RED TOMATO and a YELLOW QUINCE as possible matches. If the task is to choose which of the two possible matches is the best match with the target, one might choose the RED TOMATO, since both the apple and tomato are red and round. The pairing might be called a *surface match* because the objects appear very similar on the surface, and the classification does not depend on knowledge about apples or tomatoes. However, one might also consider choosing to pair the RED APPLE with the YELLOW QUINCE since both the target and the possible match are members of the same taxonomic family, *(Rosaceae)* and subfamily *(Maloideae)*. This pairing could be considered a *deep match*, since it deals with knowledge that is related to the phylogenetic category of the underlying objects. One would be unlikely to make the APPLE-QUINCE pairing unless one had some knowledge about their taxonomy. Moreover, if one does have this knowledge, it could overwhelm the possibility of the surface feature match. These possible pairings of course are largely influenced by the experience of the subjects (e.g., children versus adults) or the perceived goals of the subjects (e.g., a preconceived reason to classify on the basis of color versus food category).

A version of this task was used by Rabinowitz and Hogan [24] to investigate the effects of expertise in statistics. Subjects were presented with three statistics problems in a forced-choice triad task. The target matched one problem in terms of surface features and another in terms of deep (solution-related) features. Surface features included things like similar story characters and similar dependent/independent variables, while deep features included things like the kind of statistical tests needed to solve the problem (t-test, correlation, chi-square, etc.). Rabinowitz and Hogan found a positive correlation between the number of statistics courses taken (expertise) and the tendency to choose pairs that were related in terms of deep features. They concluded that more expertise in statistics generally resulted in better attention to deep, solution-relevant features of the problem. This echoes the earlier results of Chi et al. [17], and suggests that the forced-choice triad task may be sensitive to the same kinds of expertise effects as the sorting task, and may be adaptable to the study of expertise in medicine.

### The Current Research

In this paper, we suggest that physicians will demonstrate expertise effects with patient management analogous to the kinds of expertise effects found in other areas of medicine and in other domains in general. That is, we predict that expert physicians will be better able than novices to perceive and react to the deep features present in our patient profiles on the basis of similarities in management approaches. To the extent that experts perform well on classifications in which the deep-feature match deals with patient management we argue that this ability develops with relevant clinical experience rather than with explicit medical training. Put another way, we argue that physicians become experts at patient management by managing patients, not necessarily only by being in medical school.

Several predictions follow from this hypothesis. First, we predict that novice subjects should tend to make classifications on the basis of surface features. Second, in contrast to novices, we predict that expert subjects should be more likely to make matches on the basis of deep-features. Third, we expect that “patient management” will emerge as a salient category for experts and they will be able to make deep-feature matches for patients that require the same management approach. Fourth, we predict that intermediate subjects should fall somewhere in between the two extremes. That is, we predict that intermediates will make some deep-feature responses, but that they will make fewer deep-feature matches than experts. To investigate these predictions, we asked a group of endocrinologists (experts), medical residents (intermediates), and medical school students (novices) to complete a series of forced-choice triad questions designed so that one possible match was related to a deep, solution relevant feature, and the other match was related to the surface characteristics.

## Methods

### Ethics Statement

This study was approved by The University of Western Ontario Research Ethics Board for Health Sciences Research Involving Human Subjects. Informed consent was obtained from all participants. Since the study was completed online, participants read the letter of information, and their completion of the study indicated their consent to participate. This procedure was suggested, and subsequently approved, by the ethics board.

### Subjects

We tested three groups of subjects: novices, intermediates and experts. One hundred and thirty medical students from the Schulich School of Medicine & Dentistry were contacted via a mass email sent by the undergraduate office at the School on our behalf. Of the 130 students contacted, 32 started the task, and 15 completed it. Novices had not yet completed an endocrinology rotation during their training. Intermediate subjects were medical residents (postgraduate year 1 through 3) in the London Health Science Centre and St. Joseph's Health Care Hospitals in London, Ontario. Eleven residents were recruited for the study via email. Ten residents started the task and of those, eight completed it. One hundred and fifty-nine endocrinologists from Universities and hospitals across Canada were contacted via email. Experts were defined as practicing endocrinologists–no other selection criteria were used. Of the expert participants, 15 started the task and 11 completed it. Details about all three groups of subjects can be found in Table 1. We do not have complete demographic information for all the subjects who did not complete the task, so that information is not reported here.

**Table 1:** Subject Characteristics.

	N	Mean Years of Experience (range)	Training Subcategory
Expert	11	15.18 (6–24 years)	–
Intermediate	8	2.375 (2–4 years)	6 PGY1, 1 PGY2, 1 PGY3
Novice	15	0.067 (0–1 years)	5 M2, 9 M3, 1 M4
Total	36	–	–

*Note*. Clinical rotations begin in the third year of medical school, so “years of experience” begins in year 3. A fourth year student is designated one year of experience. Experts were asked how many years in practice they had (after schooling was completed), so six years were added to their responses: a physician with one year in clinical practice was assigned 7 years of experience. In the Training Subcategory column, “PGY1” refers to Post Graduate Year 1, M2 refers to Medical School Year 2, etc.

### Materials

The forced-choice task consisted of a series of ten triads, each with three hypothetical patient profiles: a target and two possible matches. We designed the task as follows: Twenty-three triads (a total of 69 profiles), including six distracter items which had no clear deep-feature match, were originally created by attending physicians who came to a consensus on the suitability of each profile individually, and each triad as a whole. In the final task, distracter items were not used because they increased the time commitment for our participants without a corresponding gain in data. Each of the remaining profiles was rated by an independent physician for readability and understandability. The rater used a scale of “1” through “7” for understandability and readability, with “1” being very hard to understand/read, and “7” being very easy to understand/read. All profiles that were used in the study were rated at least 6 for each of understandability and readability. Pilot testing with one expert, and two endocrinology fellows was used to determine which items would be used in the final task.

**Figure 1:**
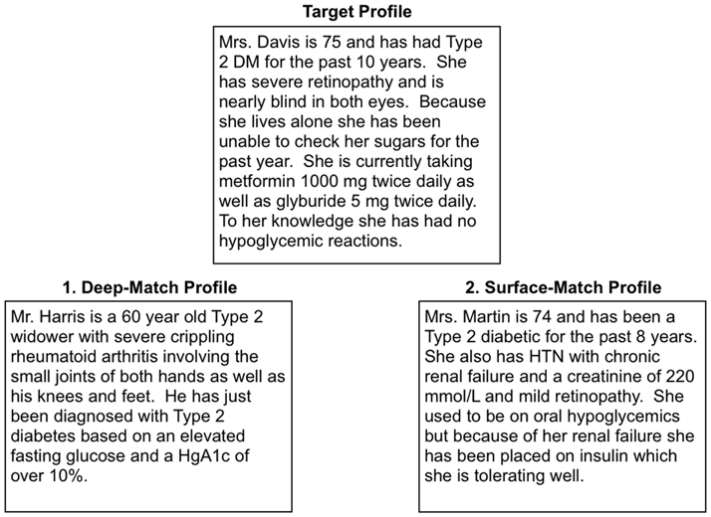
An illustration of the basic triad task. The target profile is shown at the top, and the two possible matching profiles are shown on the left and right. Profiles were shown without the “deep match” and “surface match” labels.

The triads were designed so that one profile matched the target on a number of surface features, such as patient demographics or disease history, while the other profile matched the target on deep features, such as a management approach taken to treat the patients. Half of the triads were designed so that the deep match was one that related primarily to patient management. For example, in Figure 1, the target is shown on the top, along with the potential matches (options 1 and 2). In this example, option 2 is the surface match because both patients are older females who have had diabetes for a similar number of years; they share a number of features which makes them appear similar even to non-physicians. Option 1 is the deep match because although both patients ideally should be on insulin from a biomedical standpoint, neither would be able to measure out and inject the insulin safely due to their blindness and arthritis respectively. As a result, these patients could be viewed as being in the same management category: both require a management approach that is sensitive to the patients' difficulty with home insulin administration.

The other half of the triads were designed so that to recognize the deep match, participants had to pay attention primarily to diagnostic information (e.g., diagnosing concurrent symptoms or underlying causes of diabetes; note that the primary diagnosis of diabetes was given to all participants in all profiles). Please see Appendix S1 for a complete list of all items used in the task.

### Procedure

The triad task was completed on-line by most subjects (five subjects: 3 experts, 1 resident, and 1 novice completed a paper version of the task). As described above, subjects were primarily recruited via email, which contained a link to the survey site. Subjects were asked to click the link to the triad task. Survey software was provided by the University of Western Ontario's ITS department. Demographic information, including level of schooling, number of years in practice, and proportion of patients with diabetes, was completed first. See Appendix S2 for a list of the demographic questions and possible responses.

After completing the demographic items, subjects were given instructions for the triad task.

In this survey, you will see a series of pages with 3 patient profiles on each page. Please read each profile carefully. As you read, you may notice some similarities among the three profiles. In particular, you may notice that the first profile seems to go together in a group with either Profile 2 or Profile 3. Your task is to decide whether Profile 2 or Profile 3 best forms a group with the first Profile. For each question, please indicate which profile you feel forms the best group with the First Profile. Sometimes 2 and 3 will both seem to belong in a group with the first profile; however, please choose the best pair. Then, in the space provided, please indicate why you chose that profile as a preferable match. There is no time limit on this experiment. There are also no right or wrong answers; we are simply looking for insight as to how physicians think about patients. Thank you for your participation!

The instructions were followed by ten triad questions in a random order. The same order was used for all subjects. On each triad, subjects made a selection and provided a short explanation to justify their choice. Subjects were required to submit an answer before they could move on to the next question. Once submitted, answers could not be changed. The entire process took approximately 30 minutes to complete, though there was no time limit and participants could view questions as long as they liked. In addition, subjects were permitted to log out and return to the task if needed.

## Results

### Item Analysis

To ensure suitability of each triad used, point biserial correlations were calculated for the Item-to-Test and the Item-to-Scale (either diagnosis or management, whichever sub-scale the item belonged to). A point biserial correlation is a special case of the Pearson correlation, and is used when one of the variables is dichotomous (in this case either surface or deep). This correlation indicates the relationship between performance on the item and performance on the test overall (or on the individual scale: Management or Diagnosis). For items to be valid, subjects who make deep matches should be generally more likely to make deep matches than surface matches on the task overall. Higher correlation values indicate items that are more discriminating. Highly discriminating items mean that subjects who made a deep match on an item were likely to make deep matches on the task overall, while those who made the surface match on an item were unlikely to choose deep matches on the task overall. Negative correlations indicate that people who chose deep matches on that item were likely to make more surface matches on the test overall and vice versa. Correlations significant (*p*<.05) are indicated with an asterisk in Table 2. Correlations ranged from *r_pb_* = −.23 to *r_pb_* = .81, indicating that most of the items were adequate, though the individual items varied in terms of discriminability. See Table 2 for a complete report of all correlation values.

**Table 2:** Item Analysis.

Item	Point-Biserial Correlation		Proportion Deep Responders		
	Item-to-Test (Overall)	Item-to-Scale	Novice	Intermediate	Expert
1	0.52*	0.64*	0.13	0.25	0.36
2	0.62*	0.73*	0.13	0.75	0.45
3	0.72*	0.81*	0.00	0.13	0.27
4	−0.13	0.00	0.33	0.50	0.82
5	0.19	0.25	0.20	0.75	0.55
6	0.29	0.41	0.53	0.75	0.91
7	−0.26	−0.23	0.87	1.00	0.91
8	0.53*	0.64*	0.27	0.63	0.64
9	0.43*	0.66*	0.07	0.00	0.27
10	0.20	0.30	0.47	0.38	0.45

*Note*: Correlations were significant at the *p*<.05 level are indicated with *.

The Item-to-Test correlation indicates the degree to which performance on a particular question correlates with the performance on the test overall. Item-to-Scale correlations indicate the degree to which performance on a particular question correlates with performance on its corresponding subscale (either management or diagnosis). Higher correlation values indicate that people who made a deep match on the item were likely to make deep matches on the task overall.

Proportion Deep Responders indicate the proportion of subjects in each group that made the deep response.

### Deep Feature Responding

**Figure 2:**
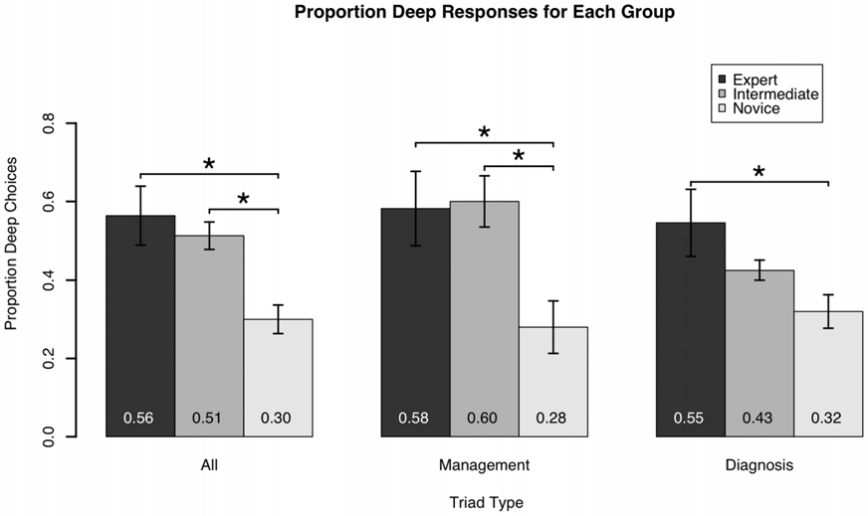
Proportion of deep-feature matches for each group of subjects. Proportions are shown for all triads (left set of bars) and for the management and diagnostic triads separately (center and right sets respectively). Significant differences at *p*<.05 are indicated with *. Error bars indicate the Standard Error of the Mean (SEM).

Our primary hypothesis concerned subjects' ability to recognize the deep-structure matches inherent in the triads. Accordingly, we calculated the average proportion-deep score for each subject across all the items. Figure 2 shows the performance by the three groups of subjects on all triads. The data show a general effect of expertise in which the experts chose the greatest proportion of deep responses, followed by the intermediates and novices. In order to analyze the overall effects, we fit a linear mixed effects model, using the proportion deep score as the dependant variable, expertise level (novice, intermediate, and expert) as a fixed effect, and question type (diagnostic or management) as a random effect within subject. The results indicated that novices' performance differed from experts', *t*(68) = −3.63, *p* = .0006, but that intermediates did not differ significantly from experts, *t*(68) = −0.24, *p* = .81. These results confirmed the existence of an expertise effect on proportion deep responding. In order to analyze this effect in greater detail, we entered the proportion-deep scores for each subject into an ANOVA with expertise as a between subjects factor. We found a significant effect of expertise, *F*(2, 31) = 8.01, *MSE* = 0.252, *p* = .002. A Tukey HSD test indicated the performance by the experts exceeded that of the novices (*M*'s = .56 and .30, *p* = .002). The performance of the intermediates (*M* = .51) also exceeded performance of the novices (*p* = .027). For the experts and intermediates, the difference in performance did not achieve significance (*p* = .810).

We also examined the proportion of deep responses by subjects for each kind of triad. Recall that half of the triads were designed so that the deep-feature match was related to patient management and half were designed so that the deep feature match was related to a diagnostic issue. If patient management is a salient category for experts, but not novices, there should be an expert-novice difference for performance on the management triads. Figure 2 also shows the data as a function of triad type, and suggests that experts performed similarly well across the two types of triads and novices were similarly unlikely to make deep feature matches. On *management* triads, an ANOVA with expertise (expert, intermediate, novice) as a between subjects factor was significant *F* (2, 31) = 5.718, *MSE* = 0.402, *p* = .008. A post hoc Tukey HSD test indicated the performance by the experts exceeded that of the novices (*M*'s = .58 and .28 respectively, *p* = .020). The performance of the intermediates (*M* = .60) also exceeded that of the novices (*p* = .026). There was no difference between expert and intermediate performance (*p* = .988). For *diagnostic* triads, a between subjects ANOVA was again significant (*F*(2, 31) = 4.08, *MSE* = 0.162, *p* = .027), with a post hoc Tukey HSD test indicating experts performed better than novices (*M*'s = .55 and .32, *p* = .020). Intermediate subjects (*M* = .43) did not differ from either the experts (*p* = .404) or the novices (*p* = .459).

Our third hypothesis involved examining the differences in subjects' performance on management versus diagnostic triads. If participants perform equally on both management and diagnostic triads, that indicates management and diagnostic information is equally salient to those participants, and that patient management is likely a category used by physicians. In order to examine this effect, we conducted a planned analysis with three paired t-tests. Each test compared the difference between diagnostic and management performance within each group of subjects. As expected, there was no difference between management and diagnostic triads for the experts, *t* (10) = 0.36, *p* = .724. There was also no difference between management and diagnostic triads for the novice subjects, *t* (14) = −0.47, *p* = .647. However, the difference between management and diagnostic triads for intermediate subjects was significant, *t* (7) = 2.50, *p* = .041.

### Correlational Analysis

We also examined the relationship between the years in practice (a reasonable measure of experience) and the tendency to choose deep-feature matches. We defined “experience” as the number of years seeing patients and we assigned the novices with the least experience (second and third year) as having 0.0 years of experience. Assigned years increased with each year starting at 1.0 for fourth-year students (because they had completed a one-year clerkship) up to the most senior physicians, who were assigned the reported number of years in practice plus six years (for their clinical training).

The data were examined separately for management trials and diagnostics trials; we found no correlation between the number of years of experience and the proportion of deep features chosen on the management triads, *r* = .17, *p* = .338. However, the correlation between the number of years of experience and the proportion of deep features chosen was relatively high on the diagnostic triads, *r* = .39, *p* = .024. These correlations are consistent with past research in medicine which has shown that years of experience is positively correlated with diagnostic performance, but not other measures of performance [28].

## Discussion

The suggestion that experts and novices would differ in their ability to classify patients was investigated using a forced-choice triad task. We found an overall effect of expertise such that experts chose the deep feature match more often than did the novice subjects. This was true both for triads in which the primary deep-feature match was diagnostic in nature and triads for which the deep feature match was related to patient management. Novice subjects, on the other hand, tended to choose matches on the basis of surface features. This suggests that the triad task was sensitive to the difference between novice and expert subjects. The tendency to make decisions on the basis of deep features is a hallmark of expert-level performance in many domains [17], [18], [24], and our results suggest that this general tendency is found in medicine as well.

Our data also suggest that patient management is a meaningful category for many expert physicians. To the extent that experts tended to make deep-feature responses more often than novices, they did so equally on diagnostic triads and management triads. That is, many of the endocrinologists in our study were experts at recognizing diagnostic similarities between patients as well the management similarities between patients. The suggestion that experts can organize knowledge around patient management is a novel contribution of the present research, and we believe that this effect has not been documented elsewhere.

However, our data also suggest that intermediates may choose deep-feature matches on the management triads—producing expert-like performance—more often than they choose deep feature matches on the diagnostic triads. This pattern was shown in our overall results in Figure 2 and by the paired t-tests analyses. This pattern of results has several possible interpretations. First, the findings may relate to the cases themselves. As we did not create matching diagnostic and management triads, it is quite possible that the diagnostic tasks simply represented a more expert knowledge base than did our management ones. A second alternative is that expertise in some aspects of clinical thinking may develop earlier in a physician's career than others. At this point our data do not distinguish between these possibilities, but we suggest that additional research is warranted to investigate category acquisition during formal medical training and early clinical experience.

### Expertise in Physicians

Our results indicate that one major prediction of our research was successful. We predicted that novice subjects should be more likely to make classifications on the basis of surface features and that expert subjects should be more likely to make classifications on the basis of deep-features. These predictions were confirmed, and the expert physicians showed cognitive effects within their area of expertise that were comparable to the kinds of cognitive effects shown by other experts [17], [20]–[22]. Our results are quite compatible with these and other studies, and suggest that one basic tenet of expert level thinking is the ability to notice and use deep, solution-relevant features.

We also predicted that experts would show a sensitivity to patient management. The notion that some physicians have a concept of patient management is novel, as most existing research on expertise and medicine has focused on diagnosis (for an excellent summary, see [4]). The current research supported our prediction. Experts did show the predicted sensitivity to deep structure for both management and diagnostic triads while novices lacked this sensitivity for both management and diagnostic triads. We conclude that physicians may indeed form categories of patients based on a specific management strategy. Future research will be needed to explore the robustness and use of these categories.

### Management and Diagnosis

Although we generally found the expected effects of expertise, our results hinted at a distinction between expertise in patient management and expertise in diagnosis. The intermediate subjects showed differential performance on management triads versus diagnostic triads: they generally performed like experts on the management triads but performed more like novices on the diagnostic triads. This was also suggested by the correlational results: the expected expertise effect was seen for diagnosis (as physicians gained more years in practice, they were more likely to choose deep matches on diagnostic triads). However, the corresponding management correlation was not seen: there was no relationship between years of experience and performance on management triads. While the correlational results are consistent with past research that shows that while age correlates with performance on diagnostic tests, it does not correlate with other measures of performance [28], this result was not quite what we had predicted. If skill in patient management were something that develops over time, we would expect a continuum of performance. However, our measure of performance, particularly at the novice level, may have been too coarse-grained to document these differences. It is possible that expertise in management might develop more rapidly (or require less experience) than expertise in diagnosis. Managing patients might not depend on the same kind of detailed, domain-specific knowledge that diagnosis does: recognition that two seemingly dissimilar patients require the same type of management is something that could begin to develop relatively early as medical students see more and more patients. Medical residents spend many hours interacting with different kinds of patients, and this might be a more general skill that residents learn. Further, this skill might be transferred to many of the rotations (e.g., cardiology, endocrinology) that residents proceed through, while the diagnostic skills they learn may be kept relatively domain specific. Another possibility, however, is that the specific items used in this task were not optimally designed for testing the intermediate subjects. The kinds of diagnostic problems faced during the final years of medical school and residency may be across a wide spectrum of problems, rather than on the narrow focus of diabetes. In other words, the intermediates may have been trained to make diagnoses, but since their training may not have been specific enough to reflect the kinds of cases we presented, they were unable to use that training effectively. Experts often rely on memory for specific examples or categories of examples when thinking [3], and most likely, the experience of the intermediates had not resulted in enough specific examples to benefit diagnostic reasoning. However, they likely had a large amount of experience interacting with a variety of patients. This may have given them enough experience in patient management to begin recognizing the deep management matches in this task.

### Additional Issues

Although our study was well controlled and resulted in compelling data about management expertise in medicine, we note that our work is still quite novel. Until now, there has been no systematic attempt to investigate expertise in goal-oriented categories like patient management in medical thinking. We feel that our work provides an important first step. However, several issues may require additional research.

First, the small sample sizes in this study is a clear limitation to generalizing the results. Second, we collected or estimated the number of years of experience for each subject, and in some cases we found that the number of years of experience correlated with the proportion of deep responses on the triad task. However, there are other ways to define and measure expertise. For example, some of the intermediates and student subjects may have performed better than others because they had a higher degree of competence. It is likely that a measure of general medical knowledge would correlate with performance in the triad task. As another example, the experts we tested may have been a heterogeneous sample in that some subjects may have had a significant patient load and others may have been involved primarily in research. It is likely that the skills developed during those two very different kinds of experience will affect performance on the triad task. In the future, it will be crucial to collect more detailed data about the kind of experience and training that subjects possess.

Third, we found effects of expertise by focusing on diabetes, which requires a high degree of management on the part of the physician and the patient. However, it is unclear if our results would generalize to other diseases or medical scenarios. For example, in many scenarios (such as the intensive care unit), physicians not only activate multiple categories while seeing a patient, they are also likely to face patients that activate competing categories. Future work should examine medical thinking and expertise in a variety of settings and should also examine triads in which the two choices would be competing deep-feature matches. This would reflect better the kinds of situations faced by physicians.

## Supporting Information

Appendix S1 List of triads used in this task.
(0.06 MB DOC)

Appendix S2 Demographic Information Collected.
(0.03 MB DOC)

## References

[1] Bordage G (1994). Elaborated knowledge: A key to successful diagnostic thinking.. Academic Medicine.

[2] Bordage G (1999). Why did I miss the diagnosis? Some cognitive explanations and educational implications.. Academic Medicine.

[3] Norman GR, Brooks LR (1997). The non-analytical basis of clinical reasoning.. Advances in Health Sciences Education.

[4] Norman GR, Eva K, Brooks L, Ericsson KA, Charness N, Feltovich PJ, Hoffman RR (2006). Expertise in Medicine and Surgery.. The Cambridge Handbook of Expertise and Expert Performance.

[5] Haynes RB, Deveraux P, Guyatt G (2002). Clinical expertise in the era of evidence-based medicine and patient choice.. Evidence Based Medicine.

[6] Barsalou LW (1983). Ad hoc categories.. Memory & Cognition.

[7] Barsalou LW (1991). Deriving categories to achieve goals.. The Psychology of Learning and Motivation.

[8] Norman GR (2005). Research in clinical reasoning: Past history and current trends.. Medical Education.

[9] Logan GD (1988). Toward an instance theory of automatization.. Psychological Review.

[10] Custers EJFM, Boshuizen HPA, Schmidt KG (1998). The role of illness scripts in the development of medical diagnostic expertise: Results from an interview study.. Cognition and Instruction.

[11] Schmidt HG, Boshuizen HPA (1993). On acquiring expertise in medicine.. Educational Psychology Review.

[12] Verkoeijen PPJL, Rikers RMJP, Schmidt HG, Van De Wiel MWJ, Kooman JP (2004). Case representation by medical experts, intermediates and novices for laboratory data presented with or without a clinical context.. Medical Education.

[13] Higgins MP, Tully MP (2005). Hospital doctors and their schemas about appropriate prescribing.. Medical Education.

[14] Freyhof H, Gruber H, Ziegler A (1992). Expertise and hierarchical knowledge representation in chess.. Psychological Research.

[15] Gobet F, Simon HA (1996). Recall of rapidly presented random chess positions is a function of skill.. Psychonomic Bulletin & Review.

[16] Simon HA, Chase WG (1973). Skill in chess.. American Scientist.

[17] Chi MTH, Feltovich PJ, Glaser R (1981). Categorization and representation of physics problems by experts and Novices.. Cognitive Science.

[18] Larkin JH, McDermott J, Simon DP, Simon HA (1980). Expert and novice performance in solving physics problems.. Science.

[19] Proffitt JB, Medin DL, Coley JD (2000). Expertise and Category-Based Induction.. Journal of Experimental Psychology: Learning, Memory, and Cognition.

[20] Medin DL, Lynch EB, Coley JD, Atran S (1997). Categorization and reasoning among tree experts: Do all roads lead to Rome?. Cognitive Psychololgy.

[21] Shafto P, Coley JD (2003). Development of categorization and reasoning in the natural world: Novices to experts, naive similarity to ecological knowledge.. Journal of Experimental Psychology: Learning, Memory, and Cognition.

[22] Johnson KE, Mervis CB (1997). Effects of varying levels of expertise on the basic level of categorization.. J Exp Psychol: Gen.

[23] Lin EL, Murphy GL (2001). Thematic relations in adults' concepts.. Journal of Experimental Psychology: General.

[24] Rabinowitz M, Hogan TM, Moore JD, Stenning K (2002). Using a Triad Judgment Task to Examine the Effect of Experience on Problem Representation in Statistics.. Proceedings of the Twenty-Third Annual Conference of the Cognitive Science Society.

[25] Rips LJ, Ortony A, Vosniadou S (1989). Similarity, typicality, and categorization.. Similarity, typicality, and categorization.

[26] Smith EE, Sloman SA (1994). Similarity- versus rule-based categorization.. Memory & Cognition.

[27] Hardiman PT, Dufresne R, Mestre JP (1989). The relation between problem categorization and problem solving among experts and novices.. Memory & Cognition.

[28] Eva KW (2002). The aging physician: Changes in cognitive processing and their impact on medical practice.. Academic Medicine.

