# Supplementary material for: Categorizing Patients in a Forced-Choice Triad Task: The Integration of Context in Patient Management
Source: PLoS One. 2009 Jun 11;4(6):e5881. doi: 10.1371/journal.pone.0005881 (PMC2690657; doi:10.1371/journal.pone.0005881)
Supplement: Appendix S1 — List of triads used in this task. (0.06 MB DOC) [file pone.0005881.s001.doc]

**Question 1**

**Target:** Mr. Clarke is a 57-year-old male who has had diabetes for the past 10 years. He lives with his wife who prepares all meals. Since losing about 20 lbs, he has been able to come off of his medications and has good sugar control. Over the past few months he has however noticed some new leg swelling. He has had no shortness of breath or chest pain and he does not wake up short of breath at night. Recent blood work showed: Na 130, K 4.5, Cl 100, HCO3 24, Creatinine 145 mmol/L, BUN 6, troponin 0.03, CK 70, HgB 125, WBC 6.1, plts 250. His HgB A1c is 6.8%. Urinalysis shows no leuks, no RBC’s, specific gravity is 1.2 and he has 3 + protein.

**Deep-feature Match:** Mr. Jackson has been a Type 1 diabetic since he was 16 years old. He is now 34. Over the past few years he has had very poor control. His diabetes has been complicated by retinopathy and nephrotic syndrome for which he is being followed by a nephrologist. He is on multiple daily injections of insulin as well as an ACE inhibitor for his nephropathy.

**Surface-feature Match:** Mr. Lewis is a 55-year-old male who has had diabetes for the past 12 years with excellent control. His only medication is metformin 500 mg twice daily. However, over the past few months he has noticed increasing shortness of breath on exertion and he wakes up at night short of breath about once/week. He has also noticed swelling of both ankles for the past month. He has had no chest pain ever. Recent blood work showed: Na 137, K 4.4, Cl 103, HCO3 22, Creatinine 95 mmol/L, BUN 8, troponin 0.03, CK 79, HgB 135, WBC 7.9, plts 275. His HgA1C is 7%. Urinalysis shows no leuks, no RBC’s, specific gravity is 1.3. His microalbumin to creatinine ratio is normal.

**Question 2**

**Target:** Mrs. Davis is 75 and has had Type 2 DM for the past 10 years. She has severe retinopathy and is nearly blind in both eyes. Because she lives alone she has been unable to check her sugars for the past year. She is currently taking metformin 1000 mg twice daily as well as glyburide 5 mg twice daily. To her knowledge she has had no hypoglycemic reactions.

**Deep-feature Match:** Mr. Harris is a 60 year old Type 2 widower with severe crippling rheumatoid arthritis involving the small joints of both hands as well as his knees and feet. He has just been diagnosed with Type 2 diabetes based on an elevated fasting glucose and a HgA1c of over 10%.

**Surface-feature Match:** Mrs. Martin is 74 and has been a Type 2 diabetic for the past 8 years. She also has HTN with chronic renal failure and a creatinine of 220 mmol/L and mild retinopathy. She used to be on oral hypoglycemics but because of her renal failure she has been placed on insulin which she is tolerating well.

**Question 3**

**Target:** Mr. Smith is a 68 year old male who has had Type 2 DM for the past 10 years. In addition to his DM he also has hypertension and high cholesterol. His sugar control has not been very good lately. He checks his sugars once a day in the morning and they are often quite high lately (usually around 18 mmol/L). His review of systems is negative except for some night sweats which he has several times per week. To his knowledge he has not had any fevers. He lives at home with his wife who prepares all of his meals. He is currently on glyburide 10 mg bid, metformin 500 mg tid and 17 units NPH insulin at supper.

**Deep-feature Match:** Mrs. Lee is a 50 year old woman of oriental descent who has had Type 2 DM for the past year. She is also suffers from chronic osteoarthritis for which she takes Tylenol ES two tablets four times/day. She checks her sugars three times per day and has recently noticed the following:AM: 2-3 mmol/L; Noon: 7-12 mmol/L; Supper: 5-7 mmol/L. For her sugars she is taking glyburide 5mg twice daily, metformin 500 mg twice daily and 15 units of NPH at bedtime. She lives alone.

**Surface-feature Match:** Mr. Nelson is a 70 year old male who has had Type 2 DM for the past 7 years. In addition to his DM he also has high cholesterol and is a smoker. His sugar control has not been very good lately. He checks his sugars three times/day and they are often quite high lately (usually around 14-18 mmol/L). His review of systems is negative except for some occasional headaches. He is currently taking glyburide 10 mg twice daily, metformin 1000 mg twice daily and 20 units of NPH insulin at supper time. He lives at home with his daughter who prepares all of his meals.

**Question 4**

**Target:** Mr. Coleman is a 65 year old man who has had diabetes for 18 years. Over the last six months he has complained of progressive fatigue and urinary frequency. He also reports losing weight. He is currently managed on metformin 2.5g/day, pioglitazone 45 mg/day and glyburide 20 mg/day.

**Deep-feature Match:** Mr. Jenkins is a 55 year old man with longstanding diabetes who develops polyarteritis nodosa leading to acute renal failure. He is managed on metformin 1.5g daily and glyburide 5 mg daily. His Cr is 300 and urea 25.

**Surface-feature Match:** Mr. Powell is a 65 year old man with diabetes and hypertension who presents with renal failure of unknown etiology. His control had been adequate and he is currently maintained on acarbose 50 mg three times daily and metformin 500 mg daily. His most recent Cr is 110 and urea 12.

**Question 5**

**Target:** Mr. Johnson is a 45 year old Type 1 diabetic with multiple complications including retinopathy for which he has had laser surgery, gastroparesis for which he is taking domperidone 10 mg with each meal and at bedtime and peripheral neuropathy for which he is on Amitriptyline 75 mg at bedtime. Over the past few months, since being switched to humalog prior to each meal, he has begun having multiple post prandial hypoglycemic episodes.

**Deep-feature Match:** Mr. Brown is 65 years old and has had Type 2 diabetes for the past 10 years. This has been complicated by retinopathy and peripheral neuropathy. He also has chronic renal failure secondary to hypertension with a creatinine of 200mmol/L. He has been on humalog for the past 3 years and has had pretty good sugar control. His wife has recently lost her job however, and he no longer has a drug plan to help pay for the humalog. He is wondering if there is a less expensive alternative.

**Surface-feature Match:** Mr. Jones is a 35 year old Type 1 diabetic who also suffers from peripheral neuropathy and retinopathy for which he has recently had laser surgery. He is on Amitriptyline 50 mg at bedtime and humalog. Since he was started on the humalog his sugar control has been excellent.

**Question 6**

**Target:** Mr. Baker is a 16 year old male who presents with a parent's complaint of difficulty losing weight. He gives a history of progressive weight gain despite numerous attempts at exercise and diet. On examination he has acanthosis nigricans and no evidence of Cushing's.

**Deep-feature Match:** Mr. Howard is a 48 year old man who presents with newly diagnosed diabetes. He has been well most of his life, but has struggled with his weight. On examination he has velvety pigmented areas on the back of his neck and under his axillae. His cardiac risk factors include an elevated LDL and low HDL

**Surface-feature Match:** Mr. Henderson is a 16 year old male who presents to the adult endocrine clinic as a transition patient. Despite good exercise, he has been gaining weight lately. He had been followed in the pediatric clinic for diabetes that was diagnosed 5 years ago.

**Question 7**

**Target:** Mr. King is a 48 year old man who presents with newly diagnosed diabetes. He has been well most of his life but over the last few years has felt progressively more fatigued. In the last year he has been diagnosed with liver failure and heart failure. He does not drink and his cholesterol profile in the past was deemed acceptable.

**Deep-feature Match:** Mr. Wright is a 48 year old man who presents with newly diagnosed diabetes. He has been well most of his life but has struggled with his weight. Over the years he has had elevated triglyceride levels and has suffered from recurrent bouts of abdominal pain. He has had recent diarrhea and complains of stool that seems difficult to flush.

**Surface-feature Match:** Mr. Adams is a 48 year old man who presents with newly diagnosed diabetes. He has been well most of his life, but has struggled with his weight. On examination he has velvety pigmented areas in the back of his neck and under his axillae. His cardiac risk factors include an elevated LDL and low HDL.

**Question 8**

**Target:** Mr. Wilson is a 50 year old construction worker. He used to have a 9-5 shift but over the past few months he has had to take whatever job he can get and the hours have not been great. He has had Type 2 DM for the past 5 years for which he takes Insulin 30/70 25 units in the morning and 15 units at supper time. Over the past 3 months he has now had at least six low sugar reactions. He now always carries a sugary snack in case this happens while he is on the job.

**Deep-feature Match:** Mr. Thomas is a 27 year old student at Fanshaw college who is studying to be a nurse. He has just started on his clinical rotations and is loving it. In addition to a single evening dose of NPH insulin at bedtime, he also takes humalog insulin with each meal.

**Surface-feature Match:** Mr. White is a 59 year old manager at Home Depot where he works in the building supplies section. He has had Type 2 DM for the past 6 years for which he takes 30/70 30 units in the morning and 10 units at supper. His control has been quite good lately. He usually has a midmorning and mid afternoon snack.

**Question 9**

**Target:** Ms. Furl is a 34 year old woman who has had Type 1 diabetes for the past 11 years. She is currently on NPH 10 units at bedtime and Humalog 6 units before each meal. She has not been self-monitoring reliably but when she has, her pre-meal readings are 10-12. Her HgA1C is 6.8%. Review of systems reveals a recent diagnosis of iron deficiency anemia secondary to poor diet and heavy periods.

**Deep-feature Match:**Mr. Trugerish is a 68 year old man who has had Type 2 diabetes for 17 years. He is currently managed with glyburide 5 mg bid, metformin 500 mg tid and rosiglitazone 8 mg daily. He checks his sugars 2-4 times per day and has recently been seeing numbers in the low teens. His HgA1C is 8.0%. Review of systems is unremarkable.

**Surface-feature Match:** Ms. Feil is a 34 year old woman who has had Type 1 diabetes for 10 years. She is currently considering going on the insulin pump. She self-monitors regularly and has been seeing numbers in the range of 4-8 mmol/L. Her HgA1C is 6.8%. Review of systems reveals that she gets occasional migraine headaches but is otherwise unremarkable.

**Question 10**

**Target:** Mrs. Bowen is a 65 year old female who has had Type 2 Diabetes for 15 years complicated by proteinuria and mild peripheral neuropathy. Over the past three years she has gained 15 lbs. She feels that this is due to progression of her osteoarthritis and reduction in her level of physical activity. She is currently managed with maximal doses of oral hypoglycemics but has poor sugar control despite this. Her most recent hemoglobin A1C was 8.5%. Although she is willing to consider being switched to insulin, she is nervous about giving herself needles.

**Deep-feature Match:** Mrs. Schneider is a 50 year old female who has recently been diagnosed with diabetes. She would like to try to control her diabetes with lifestyle modification if at all possible. At the advice of her family physician she has been trying to lose some weight but to date has been unsuccessful. She does not check her sugars at home.

**Surface-feature Match:** Mrs. Barber is a 68 year old female who has been diabetic for 9 years. Her only complication to date has been mild peripheral neuropathy. Other medical history includes osteoporosis and hypertension. She is currently on glyburide 5 mg bid, metformin 1000 mg bid and rosiglitazone 4mg OD. Her most recent hemoglobin A1C was 7%.
