# Supplementary material for: Categorizing Patients in a Forced-Choice Triad Task: The Integration of Context in Patient Management
Source: PLoS One. 2009 Jun 11;4(6):e5881. doi: 10.1371/journal.pone.0005881 (PMC2690657; doi:10.1371/journal.pone.0005881)
Supplement: Appendix S2 — Demographic Information Collected. (0.03 MB DOC) [file pone.0005881.s002.doc]

**Demographics Form**

1. What is your specialty or level of training?

– Endocrinologist

– General Internal Medicine

– Family Doctor

– Resident

– Medical Student

– Nurse

– CDE – Nurse

– CDE – Pharmacist

– CDE – Other

– Other (please describe):

2. What level of training are you at?

– M2

– M3

– M4

– PGY1

– PGY2

– PGY3

– GIM Fellow

– Endocrinology Fellow

– Family Medicine Fellow

– Other (please describe):

3. Students: During your training, have you done an endocrinology rotation?

– Yes/No

4. Clinicians:

– Number of years in practice:

– What proportion of your patients have diabetes?

5. Nurses:

– How many years of full time experience do you have?

– How many years of part time experience do you have?

– What proportion of your patients have diabetes?
